# Low-dose endotoxin inhalation in healthy volunteers - a challenge model for early clinical drug development

**DOI:** 10.1186/1471-2466-13-19

**Published:** 2013-03-28

**Authors:** Ole Janssen, Frank Schaumann, Olaf Holz, Bianca Lavae-Mokhtari, Lutz Welker, Carla Winkler, Heike Biller, Norbert Krug, Jens M Hohlfeld

**Affiliations:** 1 Department of Clinical Airway Research, Fraunhofer Institute for Toxicology and Experimental Medicine, 30625 Hannover, Germany; 2 Hannover Medical School (MHH), Hannover, Germany; 3 LungenClinic Grosshansdorf, Airway Research Center North (ARCN), Member of the German Center for Lung Research, Großhansdorf, Germany; 4 Biomedical Research in Endstage and Obstructive Lung Disease Hannover (BREATH), Member of the German Center for Lung Research, Hannover, Germany

**Keywords:** Induced sputum, Airway inflammation, Reproducibility, Sputum flow cytometry, Sputum monocytes

## Abstract

**Background:**

Inhalation of endotoxin (LPS) induces a predominantly neutrophilic airway inflammation and has been used as model to test the anti-inflammatory activity of novel drugs. In the past, a dose exceeding 15–50 μg was generally needed to induce a sufficient inflammatory response. For human studies, regulatory authorities in some countries now request the use of GMP-grade LPS, which is of limited availability. It was therefore the aim of this study to test the effect and reproducibility of a low-dose LPS challenge (20,000 E.U.; 2 μg) using a flow- and volume-controlled inhalation technique to increase LPS deposition.

**Methods:**

Two to four weeks after a baseline sputum induction, 12 non-smoking healthy volunteers inhaled LPS on three occasions, separated by at least 4 weeks. To modulate the inflammatory effect of LPS, a 5-day PDE4 inhibitor (Roflumilast) treatment preceded the last challenge. Six hours after each LPS inhalation, sputum induction was performed.

**Results:**

The low-dose LPS inhalation was well tolerated and increased the mean percentage of sputum neutrophils from 25% to 72%. After the second LPS challenge, 62% neutrophils and an increased percentage of monocytes were observed. The LPS induced influx of neutrophils and the cumulative inflammatory response compared with baseline were reproducible. Treatment with Roflumilast for 5 days did not have a significant effect on sputum composition.

**Conclusion:**

The controlled inhalation of 2 μg GMP-grade LPS is sufficient to induce a significant neutrophilic airway inflammation in healthy volunteers. Repeated low-dose LPS challenges potentially result in a small shift of the neutrophil/monocyte ratio; however, the cumulative response is reproducible, enabling the use of this model for “proof-of-concept” studies for anti-inflammatory compounds during early drug development.

**Trial registration:**

Clinicaltrials.gov: NCT01400568

## Background

Endotoxin (lipopolysaccharide, LPS) is a potent pro-inflammatory constituent of the outer membrane of Gram-negative bacteria. It occurs in a number of environments [1] and is a constituent of tobacco smoke [2] and particulate matter in indoor and outdoor aerosols [3]. Provocation of the lung with LPS induces a predominantly neutrophilic type of inflammation and has been used to study inflammatory processes. LPS challenges of the lung via inhalation or segmental application have also been used as models to test the anti-inflammatory activity of investigational new drugs [4]. The segmental application of LPS is very well controlled, as one lung segment serves as baseline, while two other segments are challenged with saline or LPS [4,5]. Only very little LPS is needed (4 ng/kg body weight) to elicit a robust influx of neutrophils or monocytes. However, the model requires repeated bronchoscopies, which limit its widespread use.

An alternative less invasive approach is LPS delivery to the lung by inhalation and the assessment of inflammation by analysis of induced sputum or exhaled nitric oxide. This has been done in healthy volunteers [6-10], in subjects with bronchial asthma [11-13], and recently in healthy smokers [14]. LPS inhalation has also been used to test the effect of salmeterol [15,16] and to compare the anti-inflammatory potential of a PDE4 inhibitor with a corticosteroid [17].

In these studies, however, rather large doses of LPS (15–50 μg) were required to induce a sufficient inflammatory response [14,17,18]. In Germany and other countries authorities now require GMP-grade LPS (manufactured under Good Manufacturing Practice standards) to be used for administration to humans. Clinical Center Reference Endotoxin provided by the NIH Clinical Center fulfills these criteria; however, it is of limited availability. Therefore, future clinical trials will need to manage LPS provocations with lower doses.

The available publications about low-dose LPS inhalation studies (< 5 μg) report either no cellular increases [6] or only minor effects [9]. In one study that showed a clear increase in sputum neutrophils after inhalation of 20,000 Endotoxin Units (i.e. 2 μg) [8], the baseline sputum induction was performed just prior to the LPS challenge, which could have enhanced the neutrophil response [19].

The only way to augment the effect of a low dose of inhaled LPS is to increase the amount of LPS that reaches the lung. It has been shown that the deposition of inhaled therapeutics can be improved by controlling the inhaled volume and the flow rate. This also reduces inter-subject variability of total particle deposition compared with uncontrolled inhalation [20]. We adopted this approach using a nebulizer with a very small dead volume (< 0.1 mL) and a computer controlled mass flow controller. After each inhalation of the LPS containing aerosol bolus, an additional air bolus was inhaled and the deposition was further enhanced by including a short end-inspiratory breath hold.

In this study we first thought to investigate whether the use of an improved inhalation procedure with a low dose of LPS elicits a sufficiently large inflammatory response, to be used in proof-of-concept studies. Secondly, we wanted to assess whether this inflammatory response is repeatable. Therefore we carried out a second LPS challenge after a four-week washout period. Finally, we tested whether a 5-day treatment with the recently approved PDE4 inhibitor Roflumilast (Daxas^®^) is able to modify the inflammatory response to LPS*.* Initially, we had planned to use a steroid for anti-inflammatory treatment (clinicaltrials.gov: NCT01400568), which is standard in the ozone challenge model [21] that also serves to induce a temporary neutrophilic airway inflammation. However, with the availability of the PDE4 inhibitor Roflumilast we decided to change to this approved COPD treatment, to include a more relevant positive control in the low-dose LPS challenge model that is planned to be used mainly in proof-of-concept studies with novel anti-inflammatory treatments developed in the field of COPD.

## Methods

### Study population

Twelve healthy, non-smoking volunteers (non-smokers for at least 5 years, history of < 1 pack year), 18–55 years old, were included in the study. The ability to produce an adequate sputum sample (≥ 1 × 10^6^ total cells, ≤ 50% neutrophils, ≤ 20% squamous epithelial cells) was tested at the baseline visit prior to inclusion. All subjects showed a normal airway response to methacholine (provocative concentration leading to a 20% fall in FEV_1_ (PC_20_FEV_1_) > 8 mg/mL). The study was approved by the Ethics Committee of the Hannover Medical School, and written informed consent was obtained from all subjects.

### Study design

**Figure 1:**
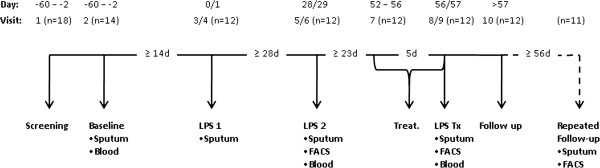
**Study design. **LPS: inhalation of 2 μg (20,000 E.U.) nebulized Lipopolysaccharide. Treat: Oral administration (500 μg/day) of the PDE-4 inhibitor Roflumilast. FACS: Flow cytometry of sputum cells was performed. In a separate study performed >56 days after the end of the LPS challenge trial, 11 subjects underwent a follow-up sputum induction. (Visits 4, 6, and 9 refer to phone calls done 24 h after the respective challenges).

**Figure 2:**
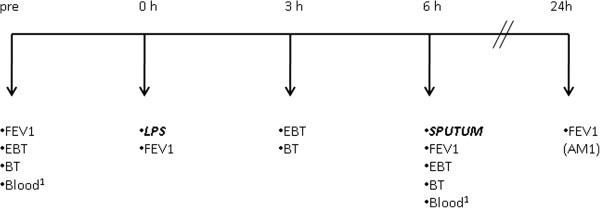
**Procedures performed on challenge days LPS 1, LPS 2 and LPS Tx (Figure **1**). **FEV_1_ = lung function measurement, EBT = exhaled breath temperature, BT = body temperature, LPS = low dose lipopolysaccharide challenge (20,000 E.U.), ^1 ^blood samples were not taken at visit 3 (LPS1). Lung function was also monitored by a portable AM1 detector hourly for 6 h, as well as 9, 11, 13, and 24 h after LPS challenge. Lung function results and the subject’s symptoms at 24 h were assessed by phone call.

This study was conducted as a non-randomized, 3-part study (Figure 1), and we included the results of a separate follow-up study to obtain data of another baseline sputum. The screening visit included documentation of the medical history and concomitant medication, an extensive medical examination including 12-lead electrocardiogram, lung function and allergy testing, a drug screening, and a pregnancy test for female subjects. Bronchial hyperresponsiveness was excluded by a methacholine challenge test. At visit 2, baseline sputum was induced and served as reference sputum for all further challenges. Challenge visits 3, 5, and 8 comprised LPS inhalation and, 6 h later, the induction of sputum (Figure 2). Blood samples were obtained before and 6 h after LPS challenge (not at visit 3). Exhaled breath- (X-halo thermometer, Delmedica) and body- (DINAMAP Pro200) temperatures were recorded prior to, 3 h, and 6 h after the LPS challenges. Lung function (FEV_1_) was measured by spirometry before, immediately after LPS inhalation, and prior to sputum induction. Pulmonary function was also monitored hourly for 6 h, as well as 9, 11, 13, and 24 h after the challenge by a portable asthma monitor (VIASYS Healthcare). A physical examination and a pregnancy test for female subjects preceded each LPS challenge, and any adverse events were recorded. Oral medication (Roflumilast, 500 μg/d) was administered for 5 days every morning including the LPS challenge day (visit 8).

All subjects except one agreed to participate in the follow-up study which included a sputum production with separate written informed consent obtained from all subjects. This visit was performed at least 8 weeks after the last LPS challenge and the data was used to further interpret the results of this study.

### LPS inhalation challenge

LPS (Clinical Center Reference Endotoxin CCRE; National Institutes of Health Clinical Center, Bethesda, USA) was dissolved in saline to a final concentration of 2 μg/mL (20,000 E.U./mL). The LPS solution was nebulized using an Aeroneb solo nebulizer (Inspiration Medical, Bochum, Germany) with a very small residual volume (< 0.1 mL). Each inhalation cycle lasted 10 seconds, using a mass-flow control unit to adjust the airflow to 150 mL/s: During the first 5 seconds, 750 mL air with nebulized LPS was inhaled, followed by 300 mL air-only over 2 seconds. An end-inspiratory breath-hold of 3 seconds completed each cycle. All subjects inhaled a total amount of 2 μg (20,000 E.U.) LPS at each challenge visit. The whole procedure lasted approximately 15 min.

### Sputum analysis

Subjects inhaled increasing concentrations of nebulized (OMRON NE-U17, Mannheim, Germany) hypertonic saline (3%, 4%, 5%) for 10 minutes each. Sputum “plugs” were selected from saliva and controlled by microscope to assure good separation from squamous cells [21]. The pooled plugs were incubated with 4 volumes of 0.1% dithiothreitol (DTT, Sputolysin; Calbiochem, La Jolla, USA) for 15 min. After adding 4 volumes of Dulbecco’s phosphate-buffered saline (DPBS; Lonza, Verviers, Belgium), the homogenized sputum sample was filtered (70 μm, BD, Heidelberg, Germany) and centrifuged (790 × g, 10 min). Total cell number and cell viability were determined with a Neubauer hemacytometer (trypan blue staining). Sputum supernatant was frozen at −80°C until analysis. For flow cytometry the cell pellet was resuspended in FACS-buffer (PBS, 5% fetal calf serum, 0.5 mM EDTA), centrifuged (790 × g, 5 min), and resuspended in FACS-buffer. Cytospots were prepared (Cytospin; Shandon, Pittsburgh, USA) and stained with Diff-Quik (Medion Diagnostics, Düdingen, Switzerland). Differential cell counts were performed by two experienced, independent observers from 400 non-squamous cells, and the results were averaged. The presented data on monocytes and small macrophages was derived from the cytospin analysis.

Cytokine concentrations in sputum supernatants were measured by ELISA, using commercial kits for both the detection of interleukin-8 (IL-8, R&D systems, Minneapolis, USA) and myeloperoxidase (MPO, BioVendor, Brno, Czech Republic). Samples were diluted 1:100 to assure cytokine concentrations within the range of the respective standard curves (limit of detection: 31.25 pg/mL for both IL-8 and MPO).

### Flow cytometry of sputum cells

An aliquot of the sputum sample was used for flow-cytometric analysis (Cytomics FC500; Beckman Coulter, Krefeld, Germany). Staining included fluorochrome-labeled antibodies from BD Biosciences (CD4 (FITC)/8 (PE), CD86 (PE-Cy7), HLA-DR (PE)) and Beckman Coulter (CD14 (APC)) and the respective non-specific isotype control antibodies from the same sources. To quantify sputum cell subpopulations, leukocytes were differentiated from cellular debris and squamous epithelial cells and further differentiated into leukocyte subpopulations by gating strategies based on light scatter properties (forward scatter: FSc, sideward scatter: SSc) and specific surface markers. To assess the expression of selected cell surface molecules such as HLA-DR and CD86 on gated macrophage populations, the mean fluorescence intensity (MFI) was measured. Specific isotype controls were subtracted from the respective MFI values. Changes (MFI difference) in the expression of these cell surface molecules were evaluated by comparing baseline and post challenge sputum cells.

### Statistical analysis

Data are displayed as arithmetic and geometric mean and standard error of the mean (SEM) or median and interquartile ranges (IQR). Repeated measures analysis of variance (ANOVA) was used to compare variables between visits. Data were log-transformed if not normally distributed. The Newman-Keuls-test was used for post-hoc analysis. Intra-class correlation coefficients (ICC) were derived from one-way ANOVA tables as the ratio of variance among subjects to total variance based on the repeated measurements [22]: (BMS-WMS/2)/((BMS-WMS/2) + WMS); BMS = between group mean square, WMS = within group mean square. A p-value < 0.05 was considered significant. For the statistical analysis we used Statistica (Statsoft, Hamburg, Germany).

## Results

### Demographics

Eighteen subjects were screened to enroll 12 subjects for the study. Three subjects were not included because of abnormal lung function or smoking history, 1 due to airway hyperresponsiveness, and 1 due to an inadequate sputum sample. One subject was screened in reserve, but inclusion was not required. Twelve subjects (3 female / 9 male) completed the study. The mean (SD) age was 38 ± 11 years and the mean FEV_1_ was 104.2 ± 7.3% predicted.

### Systemic effects of LPS

Inhalation of LPS was well tolerated with no adverse events being observed. Only a small effect on lung function was detected 1 h after LPS challenge. FEV_1_ decreased to a median (IQR) of 95.9 (9.2)% of pre-challenge values (p < 0.01). All subsequent measurements up to 24 h post LPS were not significantly different from pre-challenge values. Body temperature was slightly increased 6 h after LPS challenge (Table 1). The increase in exhaled breath temperature was even smaller, but statistically significant (ANOVA, p = 0.011, Table 1).

**Table 1:** Median (IQR) temperature (°C)

		Body **	Breath*
LPS 1	pre	36.2 (0.8)	33.8 (1.1)
	3 h post	36.2 (0.9)	33.6 (0.5)
	6 h post	36.7 (0.3)##	33.9 (0.5)
LPS 2	pre	36.4 (0.5)	33.7 (0.4)
	3 h post	36.2 (0.6)	33.7 (0.3)
	6 h post	36.6 (0.6)§	34.0 (0.5)
LPS Tx	pre	36.3 (0.6)	33.8 (0.5)
	3 h post	36.3 (0.5)	34.2 (0.7)
	6 h post	36.7 (0.3)##	33.9 (0.6)

** p < 0.01, *p < 0.05 for repeated measures ANOVA with visit (LPS1, 2, Tx) as a factor. Newman-Keuls post-hoc test: ## p < 0.01, § p = 0.08 compared with “pre”-challenge.

Compared with the screening visit we observed an increase in the median (IQR) total number of blood leukocytes (4.4 (1.6) vs. 9.5 (2.5) × 10^9^/mL) and the percentage of blood neutrophils (54.1 (9.4) vs. 74.1 (9.4)%) after LPS challenge (LPS 2). Correspondingly, the percentages of monocytes (10.4 (2.8) vs. 7.3 (1.7)%) and lymphocytes (31.9 (9.8) vs. 18.7 (7.0)%) decreased. These changes were statistically significant (ANOVA p < 0.001, each). No differences were observed in the percentage and total number of blood neutrophils and blood monocytes, when baseline and pre-challenge values were compared (baseline vs. LPS 2 vs. LPS Tx, Figure 2).

### Airway inflammation induced by low dose LPS challenge

All subjects produced adequate sputum samples throughout the study. Sputum production after LPS challenges was generally easier for subjects, as compared with the baseline sputum induction. The lower squamous cell contamination in sputum samples from these visits also indicates that sputum plugs were easier to select than in samples of the baseline visit.

**Figure 3:**
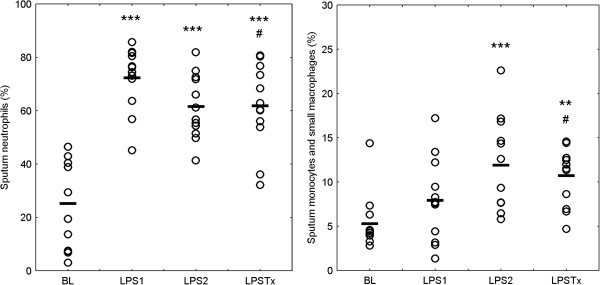
**Sputum neutrophils (left) and the sum of sputum monocytes and small macrophages (right). **Individual data and mean values of percent sputum leukocytes are displayed. BL: baseline, LPS 1: first LPS challenge, LPS 2: second LPS challenge at least 4 weeks after LPS 1, LPS Tx: third LPS challenge at least 4 weeks after LPS 2 and after 5 days of treatment with Roflumilast (500 μg /day). For statistical details please refer to Table 2. ** p < 0.01, *** p < 0.001 compared with baseline; # p < 0.05 compared with LPS 1.

Inhalation of 20,000 E.U. GMP-grade LPS induced a massive influx of neutrophils into the airways (Figure 3, Table 2). Both the increase in the percentage and in the number per mL sputum compared with baseline was statistically significant. However, the neutrophilic response to the second LPS challenge was lower compared with the first challenge. LPS also induced an influx of monocytes and small macrophages. With respect to their percentage, this effect was significant only after the second LPS challenge. The cumulative inflammatory response (sum of neutrophils, monocytes, and small macrophages; see Additional file 1: Figure S1) showed a smaller difference in percentages after the two repeated LPS challenges.

**Table 2:** Sputum composition (percentage of sputum leukocytes and cell count)

					**ANOVA**
	**Baseline**	**LPS 1**	**LPS 2**	**LPS Tx**	**p**
Macrophages (%)^§^	68.7 ± 5.3	19.1 ± 2.9***	25.9 ± 3.5***	27 ± 4.5***	<0.001
Neutrophils (%)^§^	25.2 ± 5.0	72.3 ± 3.4***	61.5 ± 3.5***	61.8 ± 4.6*** #	<0.001
Eosinophils (%)^§^	0.0 ± 0.0	0.1 ± 0.0	0.2 ± 0.1	0.1 ± 0.1	n.s.
Lymphocytes (%)^§^	0.7 ± 0.2	0.5 ± 0.2	0.5 ± 0.2	0.4 ± 0.1	n.s.
Monocytes/sm. MФ (%)^§^	5.3 ± 0.9	7.9 ± 1.4	11.9 ± 1.5*** #	10.7 ± 0.9**	0.001
Cummulative Response (%)^§^	30.5 ± 5.4	80.3 ± 3.0***	73.3 ± 3.5***	72.5 ± 4.5***	<0.001
Sq. cells (% TCC)^&^	7.7 (1.4)	2.9 (1.2)***	2.5 (1.4)***	2.8 ± 1.3***	<0.001
Total cell count (10^6^/mL)^&^	2.40 (1.2)	10.46 (1.2)***	5.08 (1.2)** ##	3.7 (1.2)*###	<0.001
Macrophages (10^6^/mL)^&^	1.43 (1.2)	1.73 (1.2)	1.11 (1.3)	0.80 (1.2)* #	0.02
Neutrophils (10^6^/mL)^&^	0.39 (1.5)	7.29 (1.3)***	2.91 (1.3)***##	2.10 (1.2)***###	<0.001
Non-Sq. epithelia cells (10^6^/mL)^&^	0.15 (1.4)	0.12 (1.5)	0.14 (1.5)	0.09 (1.4)	n.s.
Monocytes/sm. MФ (10^6^/mL)^&^	0.10 (1.3)	0.66 (1.4)***	0.52 (1.3)***	0.36 (1.2)***	<0.001
Cummulative Response (10^6^/mL)^&^	0.53 (1.4)	8.13 (1.2)***	3.47 (1.2)***##	2.51 (1.2)***###	<0.001

§ mean and standard error of the mean (SEM) of percent sputum leukocytes, & geometric mean and geometric standard error (SEM as a factor), TCC: total cell count; Sq: Squamous; sm. MФ: small macrophages; ANOVA: repeated measures ANOVA, Newman-Keuls post-hoc test: * p < 0.05, ** p < 0.01, *** p < 0.001 compared with baseline; # p < 0.05, ## p < 0.01, ### p < 0.001 compared with LPS 1. Cumulative response = Sum of neutrophils, monocytes and small macrophages.

No effects were observed for eosinophils, lymphocytes, and non-squamous epithelial cells. The total sputum cell count increased after both LPS challenges, but was lower after the second compared with the first LPS challenge. Hence, the total neutrophil count showed a significant difference between the LPS challenges, while the numbers of monocytes and small macrophages were not different.

**Figure 4:**
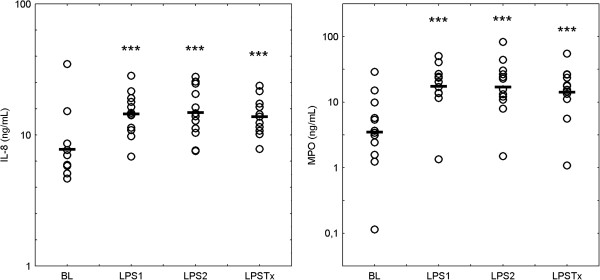
**Concentrations of IL-8 and MPO in sputum supernatants. **Individual data and mean values of log transformed values are displayed. BL: baseline, LPS 1: first LPS challenge, LPS: second LPS challenge at least 4 weeks after LPS 1, LPS Tx: third LPS challenge at least 4 weeks after LPS 2 and after 5 days of treatment with roflumilast (500 μg /day). For statistical details please refer to Table 2. *** p < 0.001 compared with baseline.

After LPS challenges, a mild increase in the sputum concentration of total protein was observed. Median (IQR) concentration at baseline, after LPS 1, LPS 2, and LPS Tx were 2.40 (0.98), 3.05 (1.26), 2.70 (0.74), and 2.78 (1.20) mg/mL, respectively (ANOVA, post-hoc test compared with baseline p < 0.05 each). Figure 4 shows the significant increases in the sputum concentration of IL-8 (ANOVA: p < 0.001) and MPO (p < 0.0005) and also illustrates that no differences between LPS 1 and LPS 2 could be observed for both these markers.

### Effect of low dose LPS challenge after treatment with Roflumilast

Prior to the third LPS challenge, all subjects were treated for 5 days with 500 μg Roflumilast/day (Daxas^®^) to test the potential modulation of the LPS response by an anti-inflammatory treatment. Only small changes in sputum composition were observed compared with the LPS challenges without treatment (Table 2). The percentage of neutrophils was significantly lower compared with the first, but not compared with the second LPS challenge. The lowest total cell count after LPS challenges was found after treatment with Roflumilast. Again, this decrease was statistically significant compared with the first LPS challenge, but not with the second. Comparable results were obtained for the numbers of neutrophils and macrophages. The inflammatory mediators IL-8 and MPO were not affected by the treatment with Roflumilast.

Flow cytometric analysis of HLA-DR and CD86 on sputum macrophages showed an increase in the MFI of these markers after treatment with Roflumilast (Additional file 1: Figures S2 and Additional file 1: Figures S3).

### Reproducibility of the LPS response

**Figure 5:**
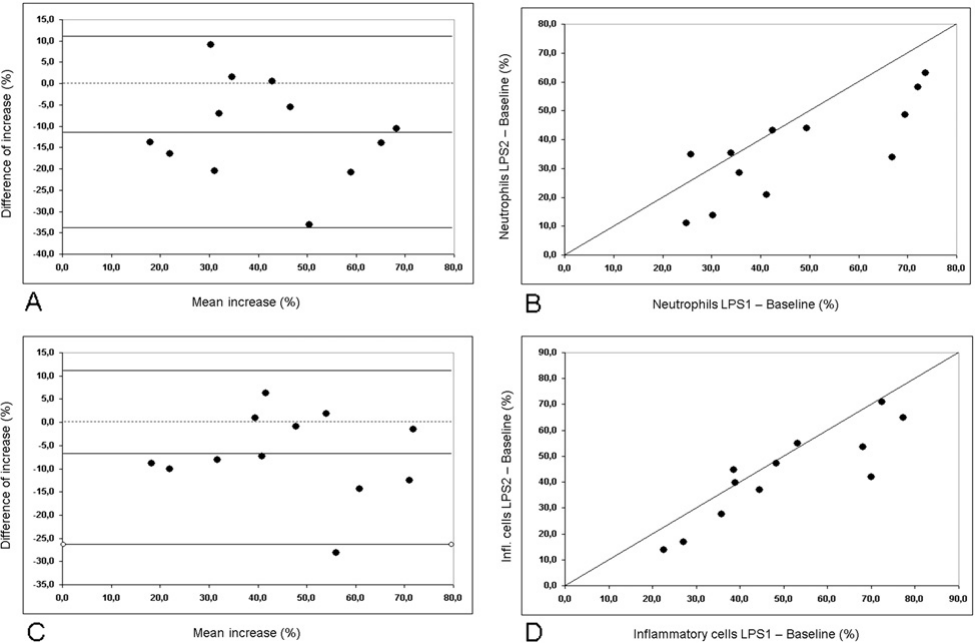
**Reproducibility of the low dose LPS induced inflammatory response (LPS 1 vs. LPS 2).** Top row (**A**, **B**): Change in the percentage of sputum neutrophils compared with baseline. Bottom row (**C**, **D**): Change in the percentage of the sum of neutrophils, monocytes and small macrophages compared with baseline. On the left (**A**, **C**) Bland-Altman plots with lines indicating the mean and 2*SD of the differences and on the right (**B**, **D**) the respective correlations with the line of identity.

In this study, the LPS challenge was performed twice to test the repeatability of the response. Compared with baseline, both provocations resulted in a significant increase in the percentage of neutrophils, but with a lower influx of neutrophils in the second challenge, as can be seen by a value for the mean difference below zero in the Bland-Altman plot (Figure 5A) and a deviation from the line of identity (Figure 5B). Despite this, there was a significant correlation between the two repeated challenges for the increase from baseline (r = 0.79). The intra-class correlation coefficient (ICC) was 0.63.

The lower proportion of neutrophils in the second challenge could partly be due to a higher proportion of monocytes/small macrophages. As LPS is well known to cause both a neutrophilic and a monocytic influx, we also analysed the cumulative response to LPS by looking at the sum of neutrophils, monocytes, and small macrophages (Figure 5C and D). Both the correlation coefficient and the ICC showed higher values for the cumulative response (r = 0.87, ICC = 0.81). We also analysed the reproducibility between LPS 2 and LPS Tx for neutrophils and the cumulative response (neutrophils: r = 0.93, ICC = 0.87, cumulative resp.: r = 0.94, ICC = 0.87).

While we observed an increase in sputum total cell count and in the cell counts of individual cell types after both LPS 1 and LPS 2, neither the cell counts nor the respective changes from baseline showed a significant correlation between the two LPS challenges (LPS 1, LPS 2).

### Sample size calculation for future studies

Based on the data of this study, we calculated the number of subjects, which would be required for a proof-of-concept study. To detect a 50% reduction of the LPS induced neutrophil level, 15 healthy subjects would need to be included. For the cumulative inflammatory response as an endpoint, the number would be reduced to 13. For a more detailed listing, refer to Additional file 1: Table S1.

### Comparison between methods

No significant relationship between any of the blood markers and the inflammation detected by induced sputum was observed. There was also no relationship between breath or body temperature and sputum markers.

The sputum slides were independently evaluated by a clinical cytologist. The differential cell counts between his results and the mean of the two independent evaluators were highly correlated (e.g. neutrophils: r = 0.89). No differences between visits with respect to scores for other morphological features (macrophages: degree of vacuolisation; epithelia cells: number and size of nuclei) were observed.

### Comparison between baseline and follow-up (unchallenged conditions)

Compared with the baseline visit of this study we observed a small but significant increase in the proportion of neutrophils (median (IQR): 19.4 (33.0)% vs. 29.6 (31.2)%, p = 0.02) in a follow-up visit, which was performed at least 57 days (max. 156 days) after the end of the last LPS challenge. This was accompanied by a slight decline in the proportion of monocytes and small macrophages (4.2 (2.4)% vs. 2.7 (2.4)%, n.s.). Neither the percentage of the sum of neutrophils, monocytes and small macrophages, nor any cell count per sputum weight showed a significant difference between these two visits. The increased levels of HLA-DR and CD86 expression on sputum macrophages observed after treatment with Roflumilast had returned to the levels observed after LPS treatment only (LPS 2).

## Discussion

Using a flow- and volume-controlled inhalation, we were able to improve the deposition of LPS in the lung and to elicit a pronounced and significant inflammatory response in healthy volunteers using a low dose of 20,000 E.U. (2 μg) LPS. When repeating the procedure after a 4 week washout period, the overall inflammatory response was shown to be reproducible; however, we did observe a small decline in the neutrophil/monocyte ratio after the second LPS challenge. Treatment with the PDE4-inhibitor Roflumilast for 5 days changed the expression of HLA-DR and CD86 on sputum macrophages, but did not result in a significant attenuation of the inflammatory cell influx. Our data suggests that the low-dose challenge required for the use of GMP-grade endotoxin is suitable for “proof-of-concept” studies of novel compounds targeting neutrophilic and monocytic airway inflammation.

Regulatory authorities increasingly control the origin and production of substances used for airway provocation. In Germany only GMP-grade LPS, such as CCRE produced by the NIH Clinical Center, is allowed to be used for these purposes. This material is also increasingly being used for studies in the USA [9,12]. As this material is of limited availability, an improved deposition and a nebulizer with a small residual volume were essential for our study. Flow controlled inhalation of nebulized aerosols can greatly improve deposition [20]. Although the AKITA^®^ inhalation system is a commercially available device with integrated flow control, we could not use this system in this study, as the death volume of its jet nebulizer is too large. Therefore we used the Aeroneb solo nebulizer (Inspiration Medical), which creates the aerosol using a high-frequency vibrating membrane with 1000 precision-cut openings, working basically like a micro-pump system. Here, the residual volume of LPS was generally below 100 μL. This nebulizer was combined with a mass-flow control unit that limited the inhalation flow and applied air only during the end of each inspiration. The controlled inhalation most likely increased the lung deposition of LPS by avoiding the unwanted deposition in the mouth and pharynx.

Inhalations of low LPS doses were safe and well tolerated. Only a small decrease in lung function was detected, but the affected subjects did not report any symptoms. The extent of systemic effects was in the expected range, with an increase in body temperature of less than 1°C. The blood total leukocyte and neutrophil count increased, but this is known to occur even after exposure to a low dose of LPS, that does not elicit a detectable change in the composition of airway leukocytes [6].

The main focus was the analysis of induced sputum. Compared with a previous study that used the same dose of LPS [9], the neutrophilic response was more pronounced. The 6 h time point after LPS inhalation has been used frequently to assess the inflammatory effect; however, there are conflicting results with respect to the maximal effect. In a study by Doyen et al., the peak neutrophil cell count was detected 24 h after the challenge [18], which is in line with data from endobronchial LPS challenges. A more pronounced effect at 6 h was recently shown by Aul and coworkers [14], but this was in healthy smokers.

Using induced sputum to assess the inflammatory response to LPS, we assessed cytospin slides after the first LPS challenge. While the neutrophil influx was clearly detectable, we also saw increased numbers of smaller macrophages and monocytes. Therefore, we included flow cytometry into our analysis of sputum composition after the second LPS challenge and measured the proportion of monocytes using CD14 staining. Comparison of the flow cytometry data with the mean cytospot cell count of two independent observers showed a good correlation for macrophages and neutrophils. We also found a fairly good relationship between CD14-positive cells and the sum of monocytes and small macrophages, supporting our approach to count these cells together (Additional file 1: Figure S4). For a more detailed flow cytometric analysis of induced sputum please refer to Lay et al. [23].

Looking at the data derived from the cytospot analysis, we observed only a small increase in the proportions of monocytes and small macrophages after the first, but a significant increase after the second LPS challenge. This effect could partly be responsible for the lower neutrophil proportion detected after the repeated LPS challenge. However, changes in cell proportions are difficult to interpret. We would, therefore, recommend using the cumulative response to LPS consisting of neutrophils, monocytes, and small macrophages as an additional outcome in future LPS trials. The reproducibility of this cumulative response compared with baseline was also better than for sputum neutrophils alone.

The development of tolerance could also be a reason, why the response to the second LPS challenge was lower. This phenomenon is well known [24-26], however, it has not been seen this clearly in a LPS inhalation trial before. Loh et al. suggested that tolerance would be visible only at low doses of LPS [27], but there are no studies available looking at repeated low-dose LPS challenges. In a recently published paper by Aul et al. [14], a dose comparable to the one used by Loh et al. was inhaled and no tolerance was detected, But Aul and colleagues challenged healthy active smokers, who are constantly exposed to LPS from cigarette smoke at relevant levels [2], and might therefore show a more homogeneous response. While this would be in favour for including only active smokers into LPS challenge proof-of-concept studies, the level of acute smoking is generally not easy to control and could bias drug effects in numerous ways. Based on our results, it appears to be advisable to include a screening LPS challenge, when planning proof-of-concept studies with healthy subjects. This was not actually tested in our trial; however, we did not see a further decline in neutrophils in the third LPS challenge, therefore, a bias due to a tolerance effect appears to be limited to the second LPS challenge in healthy subjects. In addition, the reproducibility between the second LPS challenge (LPS 2) and the LPS challenge after treatment (LPS Tx) was clearly better than between LPS 1 and LPS 2.

Interestingly, we did not see an attenuated response in the second challenge with respect to IL-8 and MPO levels in the sputum supernatant, indicating that either the sputum supernatant analysis is less sensitive or that other mechanisms than simply chemo-attraction are involved in determining the cellular response to LPS.

Comparison of sputum of 11 subjects collected on average more than 3 months (median 111, minimum 56, maximum, 157 days) after the last LPS inhalation revealed a higher mean neutrophil count as compared to the baseline visit of this study (median between baseline sputum inductions: 203 days (minimum: 191, maximum: 245). Despite this, neutrophil percentages were highly correlated (r = 0.86). It could be speculated that the reason for the increase is season-related, as baseline sputum of this study was obtained during late summer 2011 (August/September) and the repeated measurements were taken in early and colder springtime 2012 (March/April).

In this study, we investigated the effect of a 5 day treatment with Roflumilast, a duration of treatment during which a steady-state level in serum can be achieved [28,29]. In primates treated with a comparable dose (7 μg/kg body weight per day for 5 days), a small decline in BAL neutrophil numbers and percentage was observed [30]. In our study, the effect of Roflumilast treatment on sputum neutrophil percentage was small and only significant when the results after treatment were compared with the first LPS challenge. This is in line with data of COPD patients and of asthma patients after allergen challenge, obtained in two studies in which the treatment period exceeded 14 days, but did not find an effect on the percentage of sputum neutrophils [31,32]. Furthermore, Roflumilast was not able to change the relative cellular composition of BAL in healthy subjects after 4 weeks of treatment and segmental LPS challenge, while it reduced absolute neutrophil numbers [4].

With respect to the total sputum cell count and the neutrophil cell count, we observed the lowest values after Roflumilast treatment, but this decline did not reach statistical significance compared with the second LPS challenge. Based on the data in primates, we hypothesized that a 5 day treatment duration, would prove efficacious on neutrophil cell numbers. Nevertheless, the small effect on cell numbers seen in our study is compatible with the effects seen in the above mentioned studies [4,31,32]. The strongest effect on sputum neutrophil cell numbers was observed after 4 weeks of treatment. Notably, even a 4 or 2 week treatment with Roflumilast did not have profound effects on sputum inflammatory mediators, which is in line with our results. The decrease of IL-8 in COPD was borderline significant, and Roflumilast did not change sputum IL-8 and MPO after allergen challenge in asthmatic patients [31,32]. Finally, it could be speculated that in sputum, which has a higher baseline neutrophil count than BAL (approximately 20–30% compared to < 3%), there is less room for improvement of a given treatment upon endotoxin challenge and therefore effects on neutrophils are more difficult to demonstrate.

Interestingly, we found an increase in the expression of HLA-DR and CD86 on sputum macrophages. This increase was unexpected. While we can only speculate on the Roflumilast driven mechanism, we interpret this consistent finding in all subjects as an indicator for treatment compliance. Our study design was not randomized, as a clear sequence of experiments was required, in order to answer our questions. A randomized sequence would have been likely to show a larger treatment effect, but this would have been biased by an unnoticed tolerance effect.

## Conclusion

In summary, our study has shown that a low-dose of LPS, delivered by an efficient inhalation procedure, elicits a significant inflammatory response within the airways. We were also able to demonstrate that the immunological response to a low dose of LPS is complex, inducing not only the influx of neutrophils and monocytes into the airways, but potentially also involving small carryover effects and signs for the development of LPS tolerance. To utilize the advantages of an LPS model in human subjects for proof-of-concept studies, it is therefore important to assess the inflammatory response in sufficient detail, especially when working with the non-invasive method of sputum induction. We recommend including the cumulative cellular influx, the sum of neutrophils and monocytes/small macrophages, as an outcome variable. In addition, when performing studies with healthy subjects, a screening LPS challenge should be included to achieve a more homogeneous inflammatory response during the actual study period, which is then less likely to be affected by a potential tolerance bias.

## Competing interests

The authors declare that they have no competing interests.

## Authors’ contributions

OJ, BLM, CW, and LW designed, performed, analysed, and interpreted lab experiments. FS set up the inhalation system. FS and HB were the responsible medical doctors of the trial. JMH, NK, OH, and FS designed the study and wrote the study protocol. OJ, OH, and JMH drafted the manuscript. All authors read and approved the final manuscript.

## Pre-publication history

The pre-publication history for this paper can be accessed here:

http://www.biomedcentral.com/1471-2466/13/19/prepub

## Supplementary Material

Additional file 1: Figure S1 (cumulative response after LPS challenge), **Figure S2. **(flow cytometric analysis of HLA-DR and CD86), **Figure S3. **(example for flow cytometric analysis of sputum), and **Figure S4. **(correlation between microscopic and flow cytometric analysis). **Table S1.** (parameters required for sample size calculations). Click here for file
