# Supplementary material for: Low-dose endotoxin inhalation in healthy volunteers - a challenge model for early clinical drug development
Source: BMC Pulm Med. 2013 Mar 28;13:19. doi: 10.1186/1471-2466-13-19 (PMC3635929; doi:10.1186/1471-2466-13-19)

Online supplement

**Low-dose endotoxin inhalation in healthy volunteers - a challenge model for early clinical drug development**

**
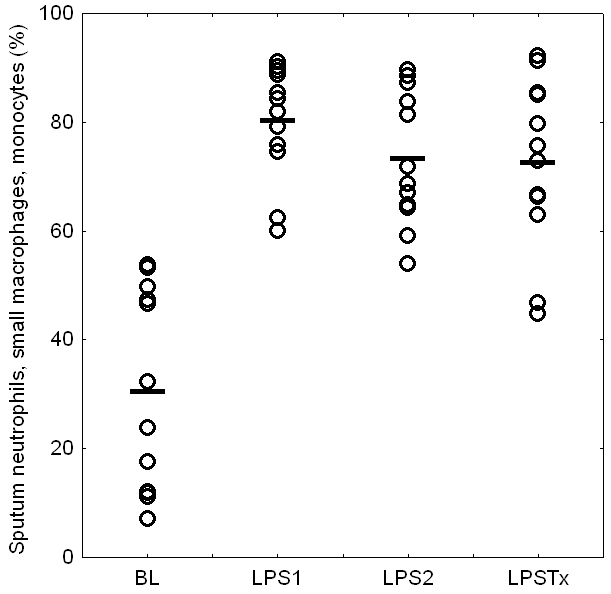
**

***

***

***

**Figure S1:** The percentage of sputum neutrophils, monocytes, small ma­cro­phages (cumulative response) 6 h after LPS challenge compared with baseline (BL). Individual data points and mean values are displayed. For statistical details please refer to the legend of table 2. *** p < 0.001.


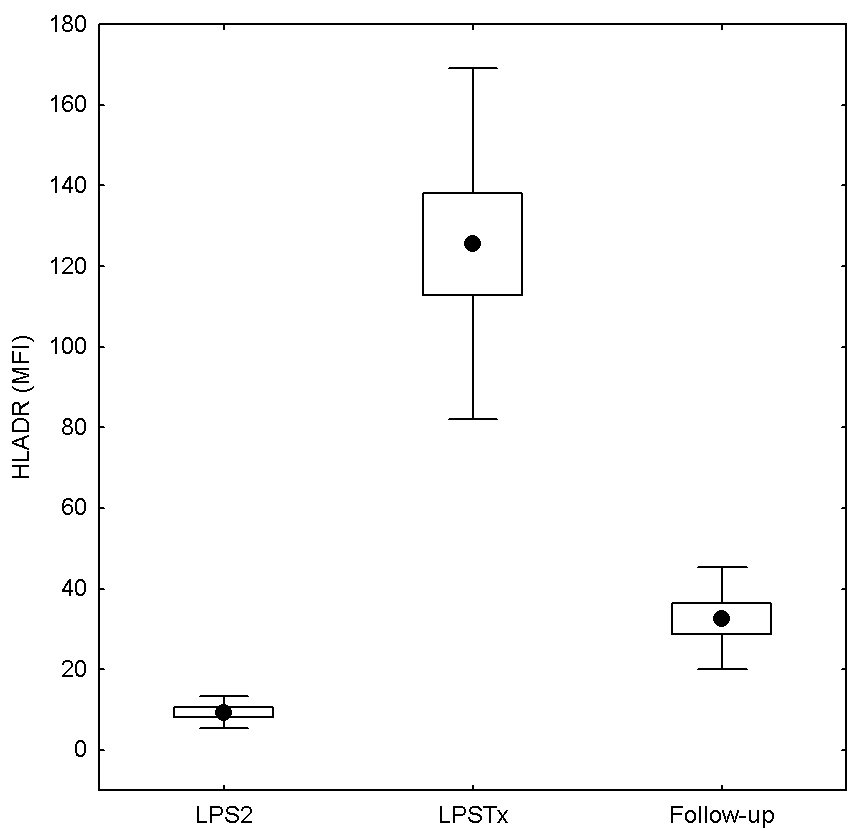

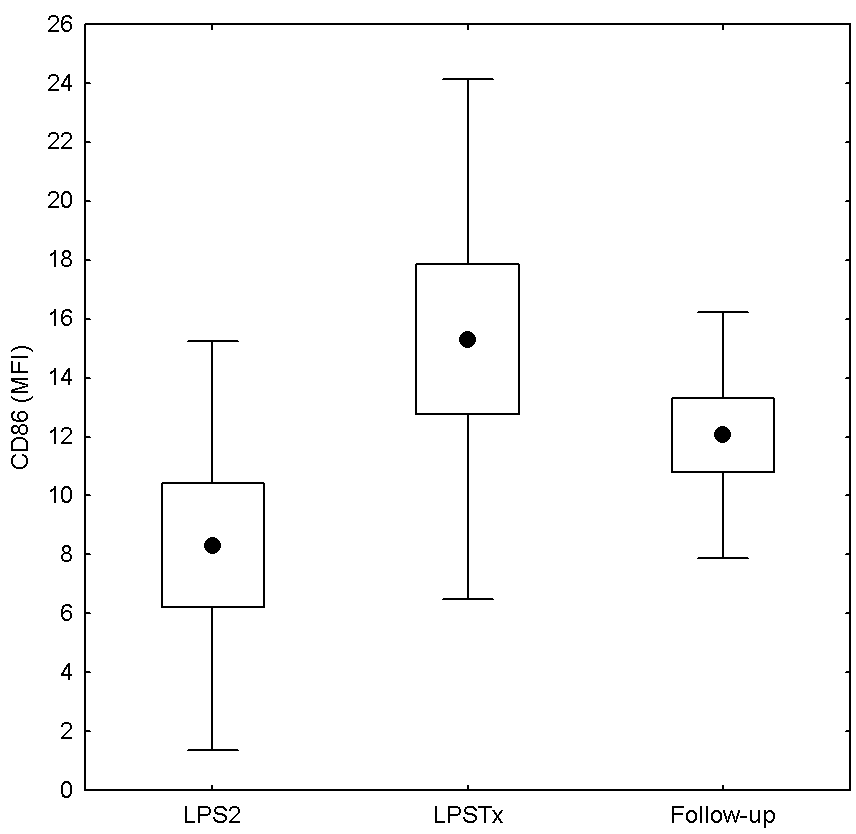


***

*

*

**Figure S2:** Flow cytometric analysis of the expression of HLA-DR and CD86 on sputum macropages. Repeated measures ANOVA HLA-DR: p<0.001; CD86: p=0.02. Post-hoc analysis compared with LPS2: *** p < 0.001, * p<0.05. Mean, SEM and SD values.


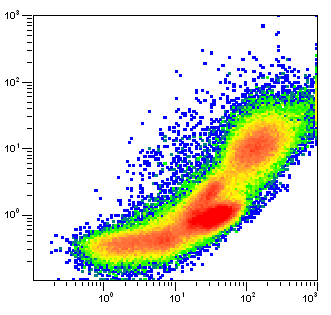

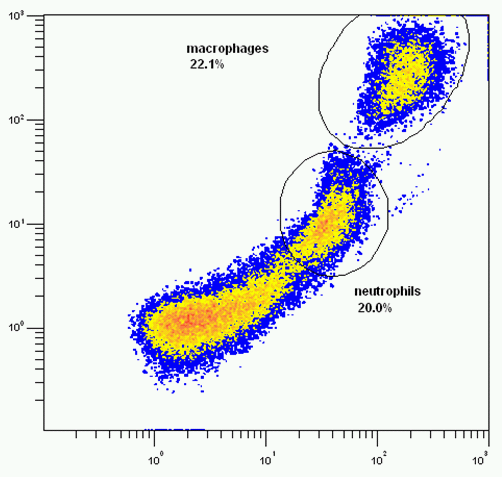


**Baseline**

**A**

**B**

**LPS 2 Isotype control**

ISO APC

CD 14 APC

SSc log

SSc log


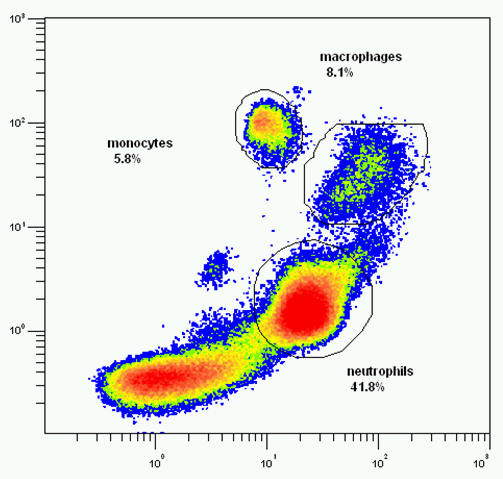

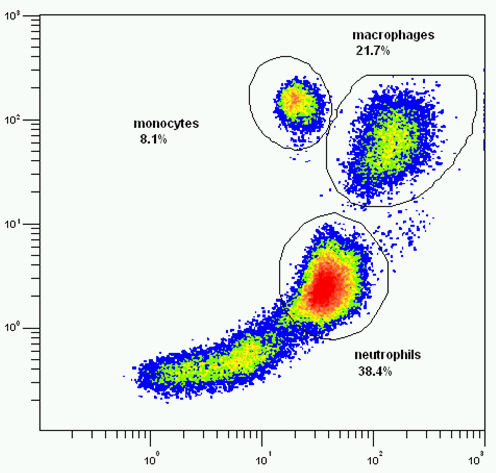


CD 14 APC

CD 14 APC

SSc log

**LPS 1**

**LPS 2**

SSc log

**C**

**D**


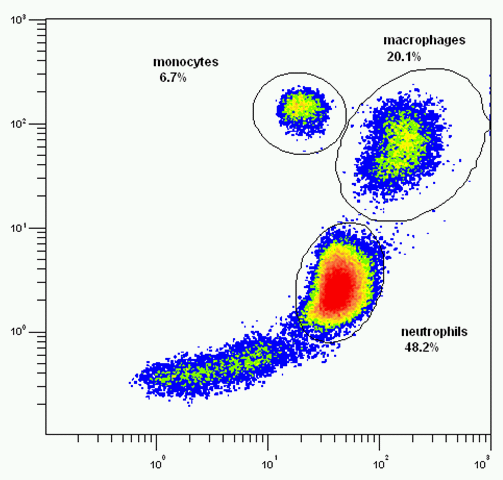


**LPS Tx**

**E**

**F**


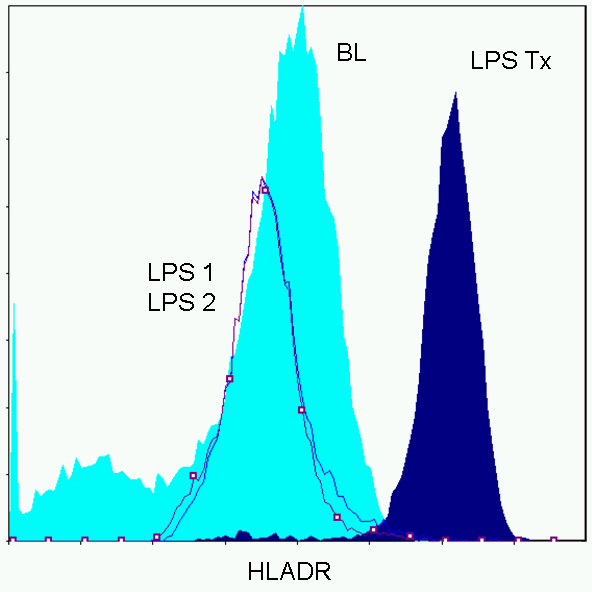


CD 14 APC

SSc log

**Figure S3:** Flow cytometric analysis of sputum monocytes and HLA-DR expression on macrophages This volunteer subjects was the only one in which sputum cells were additionally analysed by flow cytometry after LPS 1. A: Isotype control of CD14 staining. B-E: CD14 expression on macrophages and monocytes. B: baseline sputum without detectable amounts of monocytes. C: Sputum cells after the first LPS challenge LPS1. D: Sputum cells after the second LPS challenge LPS2. E: Sputum cells after the third LPS challenge LPSTx. F: Expression of HLA-DR on sputum macrophages after treatment (BL: baseline sputum, LPS 2 purple: after LPS challenge (overlaps with the expression of LPS 1blue), LPSTx: HLA-DR expression after treatment.

The parameters (means, variability) required for sample size calculations (crossover study) can be derived from table 2 of the manuscript. Here we calculate the sample size required for a proof of concept study (both to detect a reduction of the LPS induced inflammatory response of 33% or 50% by a potential novel test compound)

| **Table S1:**  **Sample size calculation** | **LPS Resp. (%)** | **SD Diff.** | **Sample size required to detect reduction of** | |
| --- | --- | --- | --- | --- |
|  |  |  | 33 % | 50 % |
| Neutrophils (LPS1-BL) | 47.1 | 18.6 | 14 | 7 |
| Neutrophils (LPS2-BL) | 36.3 | 16.2 | 18 | 9 |
| Neutrophils (LPS1/2-BL) | 41.7 | 25.8* | 32 | 15 |
| Cum. Resp. (LPS1-BL) | 49.7 | 18.5 | 13 | 7 |
| Cum. Resp. (LPS2-BL) | 42.8 | 17.5 | 15 | 8 |
| Cum. Resp. (LPS1/2-BL) | 46.3 | 25.8* | 26 | 13 |

* SD derived from one-way ANOVA (Fleiss J.L. (1986) Design and Analysis of Clinical Experiments. New York: 13 p). Sample size calculation for sputum neutrophils and for the cumulative inflammatory response (neutrophils, monocytes and small macrophages) by Statistica: One sample t-test, one-sided, Power 0.90. BL=Baseline, LPS Resp (%): Mean increase in the percentage of the respective cell type from baseline, SD Diff: SD of the mean increase from baseline, LPS1/2: here the SD was derived from both LPS1 and LPS2 challenges.


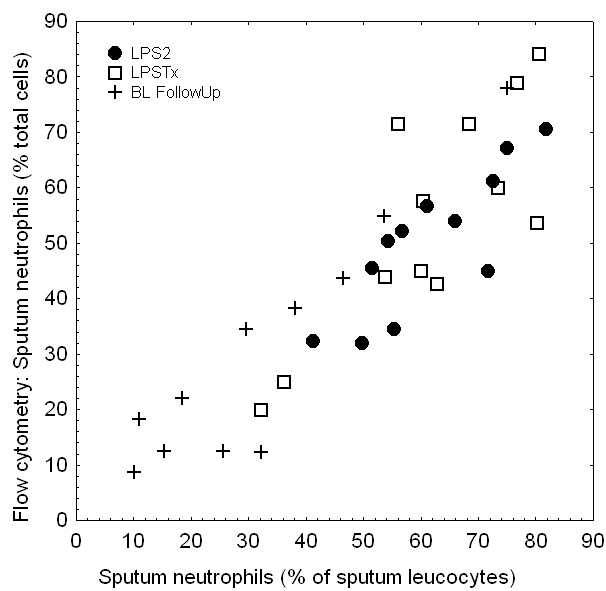

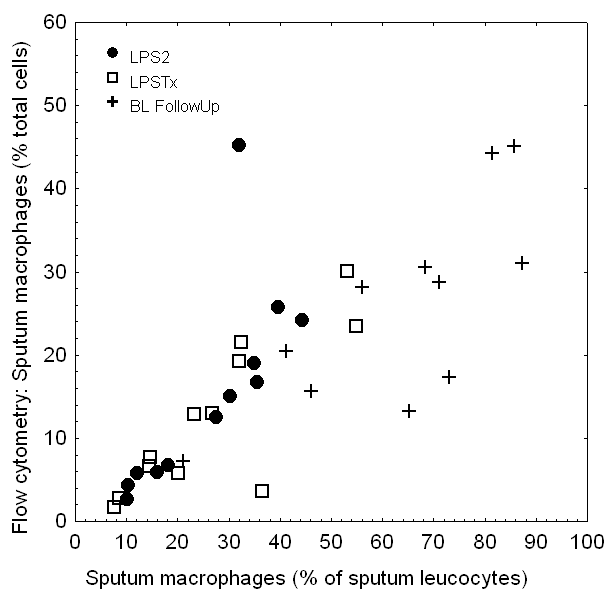


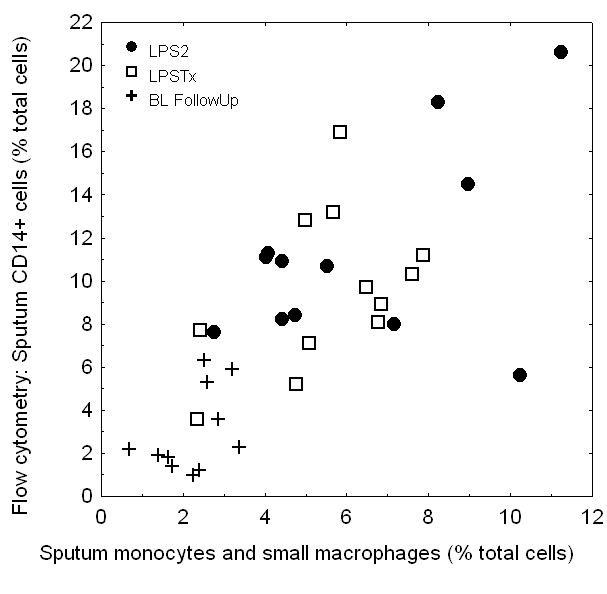


**Figure S4:** Correlation between the microscopic and flow cytometric analysis of induced sputum samples. LPS 2: second LPS challenge, LPSTx: third LPS challenge after treatment with Roflumilast, BL FollowUp: Baseline sputum obtained during a follow up visit (please refer to Study Design).


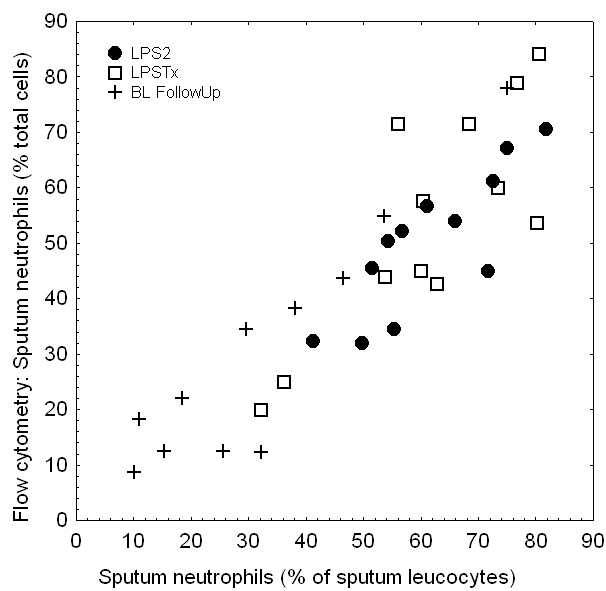

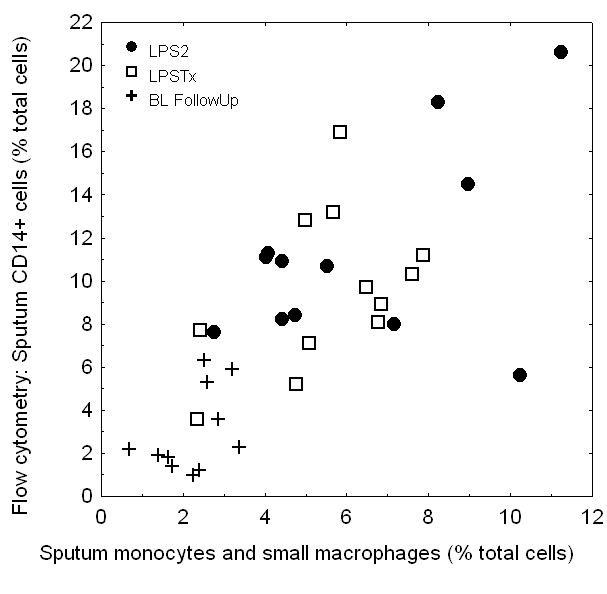

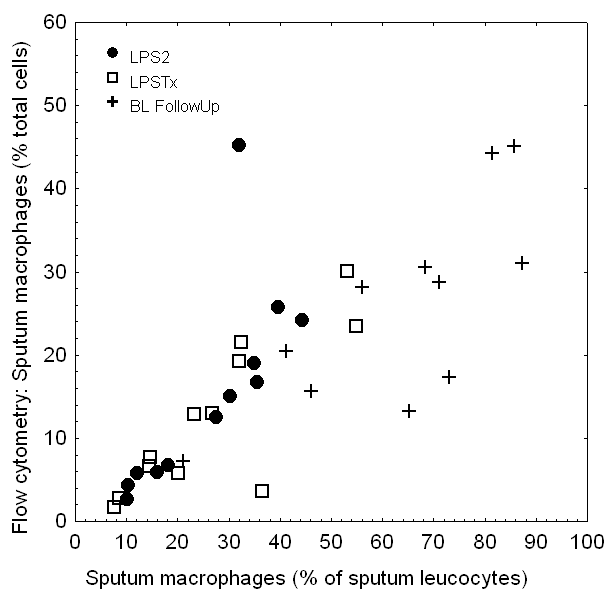

Supplement: Additional file 1: Figure S1 — (cumulative response after LPS challenge), Figure S2. (flow cytometric analysis of HLA-DR and CD86), Figure S3. (example for flow cytometric analysis of sputum), and Figure S4. (correlation between microscopic and flow cytometric analysis). Table S1. (parameters required for sample size calculations). [file 1471-2466-13-19-S1.doc]
